# Genome-Wide Assessment of DNA Methylation in Chicken Cardiac Tissue Exposed to Different Incubation Temperatures and CO_2_ Levels

**DOI:** 10.3389/fgene.2020.558189

**Published:** 2020-10-28

**Authors:** Ryan J. Corbett, Marinus F. W. te Pas, Henry van den Brand, Martien A. M. Groenen, Richard P. M. A. Crooijmans, Catherine W. Ernst, Ole Madsen

**Affiliations:** ^1^Genetics and Genome Sciences Graduate Program, Michigan State University, East Lansing, MI, United States; ^2^Animal Breeding and Genomics, Wageningen University & Research, Wageningen, Netherlands; ^3^Adaptation Physiology Group, Wageningen University & Research, Wageningen, Netherlands; ^4^Department of Animal Science, Michigan State University, East Lansing, MI, United States

**Keywords:** DNA methylation, epigenetics, temperature, CO_2_, heart, chicken

## Abstract

Temperature and CO_2_ concentration during incubation have profound effects on broiler chick development, and numerous studies have identified significant effects on hatch heart weight (HW) as a result of differences in these parameters. Early life environment has also been shown to affect broiler performance later in life; it has thus been suggested that epigenetic mechanisms may mediate long-term physiological changes induced by environmental stimuli. DNA methylation is an epigenetic modification that can confer heritable changes in gene expression. Using reduced-representation bisulfite sequencing (RRBS), we assessed DNA methylation patterns in cardiac tissue of 84 broiler hatchlings incubated at two egg shell temperatures (EST; 37.8°C and 38.9°C) and three CO_2_ concentrations (0.1%, 0.4%, and 0.8%) from day 8 of incubation onward. We assessed differential methylation between EST treatments and identified 2,175 differentially methylated (DM) CpGs (1,121 hypermethylated, 1,054 hypomethylated at 38.9° vs. 37.8°) in 269 gene promoters and 949 intragenic regions. DM genes (DMGs) were associated with heart developmental processes, including cardiomyocyte proliferation and differentiation. We identified enriched binding motifs among DM loci, including those for transcription factors associated with cell proliferation and heart development among hypomethylated CpGs that suggest increased binding ability at higher EST. We identified 9,823 DM CpGs between at least two CO_2_ treatments, with the greatest difference observed between 0.8 and 0.1% CO_2_ that disproportionately impacted genes involved in cardiac muscle development and response to low oxygen levels. Using HW measurements from the same chicks, we performed an epigenome-wide association study (EWAS) for HW, and identified 23 significantly associated CpGs, nine of which were also DM between ESTs. We found corresponding differences in transcript abundance between ESTs in three DMGs (*ABLIM2*, *PITX2*, and *THRSP*). Hypomethylation of an exonic CpG in *PITX2* at 38.9°C was associated with increased expression, and suggests increased cell proliferation in broiler hatchlings incubated at higher temperatures. Overall, these results identified numerous epigenetic associations between chick incubation factors and heart development that may manifest in long-term differences in animal performance.

## Introduction

Early life environmental parameters have profound effects on broiler chick development and post-hatch performance. Incubation temperature is one of the most important factors influencing embryonic growth, development, and physiology (Ricklefs, 1987; French, 1994; Christensen et al., 1999; Lourens et al., 2005). In studies assessing the effects of incubation egg shell temperature (EST) on organ growth, heart weight (HW) is consistently observed to be negatively correlated with EST (Leksrisompong et al., 2007; Molenaar et al., 2010, 2011; Maatjens et al., 2014, 2016). Mechanisms linking incubation EST and heart development have been proposed; studies have shown that increased incubation temperature decreases the mitotic index of cardiomyocytes (Romanoff, 1960), as well as the concentration of circulating T3, the metabolically active form of thyroid hormone (Kühn et al., 1984; Yahav et al., 1996). Additionally, phenotypic differences in adult broilers associated with differences in early life temperature have been observed. Chicks incubated at a higher EST from embryonic day 7 (E7) to hatch were found to have a higher incidence of ascites mortality at 6 weeks of age (Molenaar et al., 2011; Sözcü and Ipek, 2015). However, broilers exposed to increased temperature early in life exhibited decreased mortality when exposed to heat stress again at 6 weeks of age relative to unexposed animals, suggesting that early heat stress confers thermotolerance that can improve performance upon subsequent exposures (Yahav and Hurwitz, 1996).

In addition to temperature, CO_2_ concentration during the incubation period has been suggested to be important for regulating chick growth and physiology. Embryos incubated at increased CO_2_ concentration have been found to exhibit numerous phenotypes; in addition to a significantly greater average HW at hatch (Maatjens et al., 2014), lower blood oxygen levels relative to control chicks have also been observed (Hassanzadeh et al., 2010). Increased incubator CO_2_ concentration was associated with decreased ascites mortality in broilers later in life (Buys et al., 1998), suggesting long-term effects of early life CO_2_ exposure. Alterations made to the epigenome have been proposed as one mechanism by which early life environmental differences can manifest in phenotypic differences in adult precocial birds (Tzschentke and Basta, 2002; Nichelmann, 2004), and it has recently been shown that embryonic thermal manipulation induces epigenetic modifications in the hypothalamus of 35-day old broilers (David et al., 2019).

Numerous epigenetic molecular mechanisms including DNA methylation, histone tail modifications, and non-coding RNA interactions have been observed to induce mitotically heritable changes in gene expression without altering DNA sequence (Jaenisch and Bird, 2003; Ibeagha-Awemu and Zhao, 2015). DNA methylation is an epigenetic modification involving the enzymatic addition of a methyl group to the 5-carbon of cytosine rings, producing 5-methylcytosine. Methylation occurs almost exclusively at CpG dinucleotides in vertebrates and has context-specific associations with gene expression. In gene promoters, methylation generally functions to decrease levels of transcription through the alteration of transcription factor (TF) binding sites, the recruitment of transcriptional repressors, or changes in chromatin conformation (Bird and Wolffe, 1999; Greenberg and Bourc’his, 2019). Conversely, gene body methylation is generally associated with increased levels of transcription (Lorincz et al., 2004; Ball et al., 2009; Maunakea et al., 2010), although negative associations have been identified in the context of genic enhancers (Varley et al., 2013). Epigenetic processes are known to be altered in response to environmental perturbations, and this phenomenon has been studied in livestock species in response to heat stress (Hao et al., 2016; Skibiel et al., 2018; Vinoth et al., 2018). Additionally, numerous epigenome-wide association studies (EWAS) have been performed in humans and mice to identify loci at which methylation level is significantly associated with complex and disease traits (Orozco et al., 2015; reviewed in Flanagan, 2015). Bisulfite-sequencing approaches allow for genome-wide assessment of DNA methylation patterns, yet this approach has been underutilized in the chicken thus far (Li et al., 2015; Lee et al., 2017; Zhang et al., 2017, 2018).

In the current study, we assessed DNA methylation patterns in cardiac tissue of broiler chicks incubated at normal or high EST (37.8 or 38.9°C, respectively) and low, medium, or high CO_2_ concentration (0.1%, 0.4%, 0.8%, respectively) from embryonic day 8 (E8) until hatch. Using reduced representation bisulfite sequencing (RRBS), we tested for differences in CpG methylation rates between EST and CO_2_ treatments and identified thousands of loci exhibiting differential methylation that are associated with heart development. Additionally, by integrating phenotypic records from the same samples, we performed an EWAS and identified loci at which methylation rate was significantly associated with HW, and many of these sites were within genes with known roles in heart development. Our results provide evidence that EST and CO_2_ may be influencing heart growth and physiology through changes in cardiac DNA methylation patterns.

## Materials and Methods

### Experimental Design and Sample Collection

Heart tissue samples were collected from a 2 × 3 factorial study. Briefly, Cobb 500 broiler eggs were obtained from commercial broiler breeder farms and incubated in six consecutive batches. From day 0 to 8 of incubation (E8), all eggs were incubated at an EST of 37.8°C and 0.1% CO_2_. At E8 eggs were divided into two ESTs (37.8°C and 38.9°C) and three CO_2_ concentrations (0.1%, 0.4%, and 0.8% CO_2_) until hatch. Both EST treatments were applied in all batches, but CO_2_ treatment application varied between batches. Time until hatch was recorded for each chick, and animals were removed from the incubator 6 h post-hatch. Chicks were then killed by cervical dislocation followed by decapitation. Hearts were removed and stored at −20°C until further analysis. Heart samples derived from the left ventricle of 84 chicks (13 to 15 samples per EST-CO_2_ combination) were utilized for DNA isolation and RRBS.

### Statistical Analyses of Heart Weight

We tested for the significance of EST, CO_2_ concentration, and their interaction on HW using analysis of variance (ANOVA). To account for the significantly shorter incubation time of 38.9°C chicks, we constructed a linear model with HW as the response variable and incubation time as a fixed effect, and subsequently used the model residuals as a response variable to test the significance of the environmental parameter main effects and their interaction.

### Sample Processing and Bisulfite Sequencing

DNA and RNA from heart samples were isolated using the AllPrep DNA/RNA Mini Kit (Qiagen) following manufacturer’s instructions. The RRBS libraries were prepared with the Ovation RRBS Library construction kit from NuGEN following manufacturer’s instructions. Isolated DNA was cut with *Msp*I and a fragment size selection of 20–250 bases was applied. Samples were pooled across 9 flow cell lanes with each pool containing no more than 10 samples, and all pools were sequenced for 161 cycles on a HiSeq 2500 using the TruSeq SBS sequencing kit version 4. Fastq files were generated and demultiplexed with the bcl2fastq v2.17.1.14 Conversion Software (Illumina).

### Bioinformatics Analyses

Raw RRBS FASTQ files were trimmed of adapter sequences using Trim Galore v0.5.0 with Cutadapt v1.8.1 (Babraham Bioinformatics) and the parameters: -a AGATCGGAAGAGC. Reads were subsequently filtered for only those beginning with the expected YGG trinucleotide sequence using a python script provided by NuGEN Technologies^1^.

Read alignment was performed using BS-seeker2 v2.1.3 (Guo et al., 2013). An RRBS bowtie2 index was generated from the *Gallus gallus* GRCg6a reference genome assembly using bs_seeker_build.py with the following parameters: –aligner bowtie2 –rrbs –low 10 –up 280. Trimmed reads were subsequently aligned to the RRBS index using bs_seeker2-align.py with the following parameters: –rbbs –low 10 –up 280 –mismatches 0.05 –aligner = bowtie2 –bt2-p 10 –bt–local –bt2-N 1. Binary alignment map (BAM) files were converted to CGmap files using CGmapTools v0.1.2 (Guo et al., 2018), which reports methylation rates for all covered CpGs.

CGmap files were used to calculate sample global methylation rates, and to assess their associations with EST, CO_2_, and individual CpG methylation rates. We identified an overrepresentation of CpG sites where methylation was significantly correlated with sample global CpG methylation rate (data not shown). We therefore corrected for this factor in subsequent site-specific differential methylation analyses.

### Differential Methylation Analyses

We filtered CGmap files to only include CpGs with coverage of at least 10 reads within a sample. Filtered CGmap files were subsequently converted to CpG report text files for *methylKit* analysis using a custom python script.

Differential methylation analyses were performed using the *methylKit* R package v1.8.1 (Akalin et al., 2012). Briefly, we removed CpGs in the 99.5th percentile of coverage for each sample to remove potential PCR duplicates. For the EST differential methylation analysis (38.9 vs. 37.8°C), CpGs were retained if they were covered in at least 25 samples per EST treatment over all CO_2_ treatments. A logistic regression model was fitted for each CpG accounting for the covariates of CO_2_ level and sample mean CpG methylation rate to test if EST had a significant effect on the log odds ratio of the CpG methylation rate. For CO_2_ differential methylation analyses, CpGs were retained if they were covered in at least 16 samples per CO_2_ treatment over both EST treatments. Three different analyses were performed to contrast the three different CO_2_ treatment pairs (0.4 vs. 0.1%, 0.8 vs. 0.4%, and 0.8 vs. 0.1% CO_2_). Again, a logistic regression model was fitted for each CpG, accounting for covariates of EST and sample mean CpG methylation rate, and testing for the effect of CO_2_ level. A CpG site was considered significantly differentially methylated (DM) if the difference in methylation rate between treatments was greater than 10% and the corresponding *q*-value was less than 0.05 in both EST and CO_2_ comparisons.

### CpG Annotation

All analyzed CpGs were annotated with respect to overlap with genes using the genomation R package v1.14.0 (Akalin et al., 2015). We defined promoter CpGs as those within 2 kb upstream or 200 bp downstream of a gene transcription start site (TSS). Intragenic CpGs were defined as CpGs within any other region of a gene, while all remaining CpGs were classified as intergenic. Promoter- and intragenic-DM genes (DMGs) were defined as those with a DM promoter and intragenic CpG, respectively.

To determine if DMGs were disproportionately involved in certain biological processes, gene lists were submitted for gene set enrichment analysis (GSEA) using the Panther database (Thomas et al., 2003, 2006). We submitted promoter- and intragenic-DMG lists separately, and a background list composed of all genes with CpGs covered by our RRBS data was applied. We considered GO terms significantly enriched at FDR < 0.05.

### Motif Enrichment Analysis

We extracted 100 bp genomic sequences centered around EST hyper- and hypomethylated CpGs, as well as 100 bp sequences at 10 k random CpGs included in the DM analysis to use as control sequences. Sequences were uploaded to the MEME suite (Bailey et al., 2009; McLeay and Bailey, 2010) and the Analysis of Motif Enrichment (AME) tool was applied to identify enriched motifs in the Vertebrates (*in vivo* and *in silico*) database among our hyper- and hypomethylated sequences. We utilized the average odds score scoring method and Fisher’s exact test to calculate motif enrichment. Motifs were considered significantly enriched at adjusted *p*-value < 0.05 and *E*-value < 0.05. We identified motifs that were uniquely enriched among hyper- or hypomethylated sequences, as well as their corresponding TF. The genes encoding these TFs were submitted to GSEA using the PANTHER database to identify enriched GO terms.

### Epigenome-Wide Association Study

We performed an EWAS to identify significant associations between CpG methylation rates and HW. CpG sites with a methylation rate variance of at least 1 across samples with coverage ≥10 were retained for further analysis. For each CpG site, a linear model was fitted with methylation rate as a response variable and including the fixed effects of sample mean methylation rate and CO_2_ level. Residual methylation rates were extracted and used for subsequent association analyses. For each CpG site, a linear model was fitted with log2-transformed HW as a response variable, and CO_2_ concentration, sample mean methylation rate, and CpG methylation rate as fixed effects. Analysis of variance was performed to assess the effect of the CpG methylation rate on HW, and this process was repeated for all sites. Associations were considered significant at *p* < 1e-5.

### Quantitative Reverse-Transcription PCR

We performed quantitative reverse-transcription PCR (RT-qPCR) on eight DMGs (*TBX1*, *TBX4*, *THRSP*, *PITX2*, *ABLIM2*, *NGB*, *GSC*, and *DAG1*). Total RNA from six samples per EST treatment was reverse transcribed using Superscript II Reverse Transcriptase (Invitrogen). Custom primers were designed for the eight DMGs and two stably methylated genes (*MEAF6* and *SRRM1*), as well as for *YWHAZ* and *RPL13* which have been cited as suitable reference genes in chicken heart (Hassanpour et al., 2018; Supplementary Table S1). All assays were performed in singleton on a QuantStudio 5 Real-Time PCR System (Thermo Fisher) using 3.75 ul cDNA template (10 ng total), 1.25 ul of forward and reverse primers, and 6.25 ul MESA BLUE qPCR Master Mix (Eurogentec). Cycling conditions were 50°C for 2 min. and 95°C for 10 min., followed by 40 cycles of 95°C for 15 s and 60°C for 1 min., followed by a melt curve stage of 95°C for 15 s and 60°C for 1 min. and a dissociation step of 95°C for 15 s. Delta Cts (dCts) were obtained for each gene per sample by subtracting the test gene Ct from the geometric mean of the reference gene Cts, and analyses of variance were performed to assess the significance of EST on dCts correcting for sample CO_2_ concentrations. Fold changes in abundance at 38.9°C relative to 37.8°C were calculated using the 2^-ddCt method.

## Results

### Broiler Chicks Differ Significantly in HW Between ESTs

**FIGURE 1:**
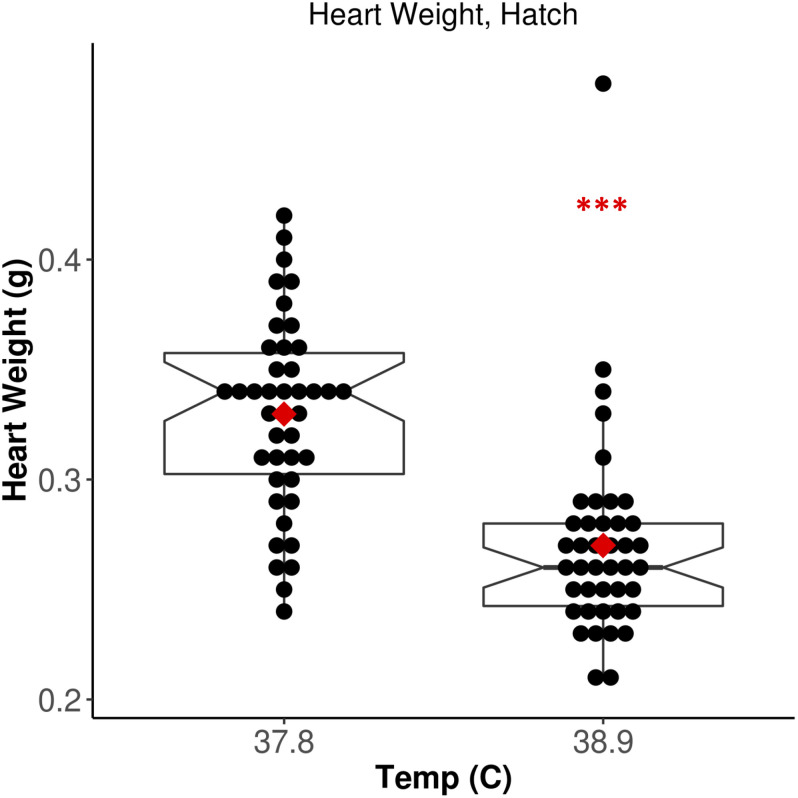
Increased EST is associated with significantly lower heart weight. Notched horizontal lines and red dots indicate treatment median and mean values, respectively. ***p < 0.001.

We measured HW in all broiler chicks 6 h post-hatch. Chicks incubated at 38.9°C exhibited significantly lower HW relative to 37.8°C chicks (Figure 1; *F* = 37.53, *p* = 3.19E-08). There was no significant effect of CO_2_ concentration (*F* = 0.254, *p* = 0.62) or EST-CO_2_ interaction (*F* = 0.257, *p* = 0.61) on HW. When correcting for the significantly shorter incubation time of 38.9°C chicks (*F* = 52.79, *p* = 2.18E-10), the difference in HW between ESTs remained significant (*F* = 12.75, *p* = 6.05E-04). These results recapitulate the well-established negative association between incubation EST and HW that has been found in numerous studies (Leksrisompong et al., 2007; Molenaar et al., 2010, 2011; Maatjens et al., 2014, 2016), and provided strong support for the assessment of associated differences in cardiac methylation between ESTs.

### RRBS Reads Capture Predominantly Lowly Methylated Regions of the Chicken Genome

An average of 18.9 million RRBS reads were generated per sample, of which 18.3 million remained after trimming (Table 1). Mapping of RRBS reads to the *G. gallus* reference genome resulted in an average unique alignment rate of 73.8%, and an average of 3.1 million CpGs covered at a read depth ≥10X. Global CpG methylation rates across all samples were low on average (17.7%), likely due in part to the fact that RRBS targets CpG-rich regions of the genome which are lowly methylated (Meissner et al., 2008). Average CHG and CHH methylation rates were 0.55% and 0.56%, respectively; given the inherently low prevalence of methylation in non-CpG contexts in vertebrates (Ziller et al., 2011), this suggests high bisulfite conversion efficiency of sequenced reads across samples.

**TABLE 1:** Summary of RRBS mapping.

	**No. Raw Reads**	**No. Trimmed Reads**	**Unique Mapping Rate (%)**	**CpG Methylation Rate (%)**	**CHG Methylation Rate (%)**	**CHH Methylation Rate (%)**	**CpGs Covered**
**Mean**	18.9 M	18.3	73.8	17.7	0.55	0.56	3.1 M
**Range**	10.1–34.1 M	9.6–32.8 M	77.1–69.9	13.8–20.4	0.48–0.78	0.49–0.77	2.3–3.5 M

A total of 1,874,588 CpG sites were shared across at least 25 samples per EST treatment, and the majority of these (40.56%) were within gene promoters. 35.18% of CpGs were within gene bodies (25.67% in introns and 12.51% in exons), while 21.26% were intergenic. Promoter CpGs exhibited the lowest methylation rate on average (7.76%), with 85% of sites having an average methylation rate less than 10% (Supplementary Figure S1A). The percentage of lowly methylated (<10%) CpGs in exons, introns, and intergenic regions was significantly lower – 42.74%, 54.52%, and 51.77%, respectively (Supplementary Figures S1B–D). The low methylation observed at a majority of CpGs indicates that many loci covered by our RRBS data are not dynamically methylated in response to the applied environmental conditions, but remain relatively unmethylated regardless of EST or CO_2_ treatment.

### EST Impacts the Methylation State of Genes Involved in General and Heart-Specific Developmental Processes

**FIGURE 2:**
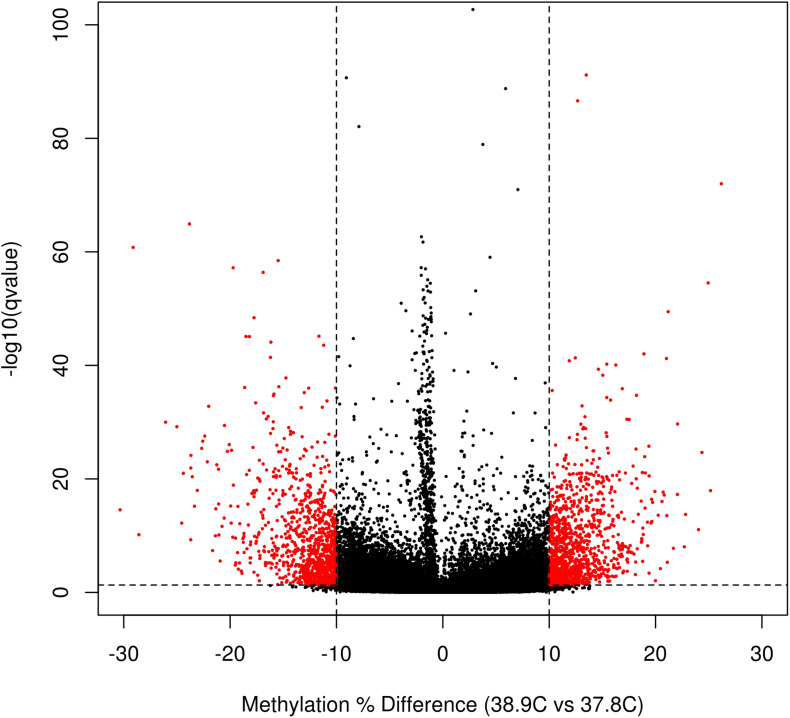
Volcano plot of CpG differential methylation between incubation ESTs. Horizontal line indicates the *q*-value threshold for significance (0.05), and vertical lines indicate methylation difference threshold for significance (10% difference between EST treatments).

We identified a total of 2,175 DM CpGs between EST treatments, of which 1121 were hypermethylated and 1054 hypomethylated at 38.9°C relative to 37.8°C (Figure 2 and Supplementary Table S2). Differential methylation was more likely to occur outside of promoter regions; of the 760,332 promoter CpGs tested, only 285 (0.05%) were DM, while 0.14%, 0.18%, and 0.15% of exonic, intronic, and intergenic CpGs, respectively, were DM. In total 269 genes were promoter-DM (promoter-DMGs; 123 hyper- and 146 hypomethylated), while 949 genes were intragenic-DM (intragenic-DMGs; 526 hyper- and 423 hypomethylated).

DM gene lists were submitted to Panther for GSEA, and we identified uniquely enriched biological processes among different gene sets (Supplementary Table S3). We did not identify enriched GO terms when considering hyper- and hypomethylated promoter-DMGs separately, however, when combining the two groups we identified enrichment of the terms “Multicellular Organism Development,” “Animal Organ Development,” and “Anatomical Structure Development” (Table 2). Among promoter-DMGs, several are known to play roles in heart development, including T-box transcription factors 1 and 4 (*TBX1* and *TBX4*) that were hypomethylated at 38.9°C, and two genes involved in thyroid hormone signaling: thyroid hormone receptor alpha (*THRA*) and thyroid hormone responsive (*THRSP*), which were hypo- and hypermethylated, respectively (Supplementary Figures S2A,B). As promoter methylation is generally inversely correlated with gene expression, hypomethylation of these gene promoters at 38.9°C is indicative of increased potential for gene activation, while hypermethylation suggests decreased activation.

**TABLE 2:** Enriched GO terms among DMGs between EST treatments.

**GO term**	**No. Genes**	**Enrichment**	***P*-value**
**Promoter-DMGs**			
anatomical structure development	86	1.53	1.19E-05
animal organ development	60	1.81	4.22E-06
multicellular organism development	84	1.61	2.06E-06
**Intragenic hypermethylated genes**			
anatomical structure development	179	1.82	1.93E-18
cell differentiation	131	1.95	3.15E-11
cardiovascular system development	29	3.04	2.71E-07
negative regulation of myoblast differentiation	5	10.21	2.37E-04
heart development	22	2.30	4.33E-04
regulation of cardiac muscle tissue development	7	4.82	9.30E-04
**Intragenic hypomethylated genes**			
anatomical structure development	165	1.97	2.32E-21
cell differentiation	111	1.93	1.65E-12
cell adhesion	36	2.53	8.86E-07
positive regulation of transcription	50	2.08	1.20E-06
glycosaminoglycan metabolic process	11	4.64	4.52E-05

Intragenic-DMGs were enriched for processes related to cardiac muscle development (Table 2). Specifically, hypermethylated intragenic-DMGs were enriched for GO terms such as “Heart Development,” “Regulation of Myoblast Differentiation,” and “Cardiovascular System Development,” while hypomethylated intragenic-DMGs were enriched for terms including “Cell Differentiation,” “Cell Adhesion,” and “Glycosaminoglycan Metabolic Process.” Among genes associated with these GO terms are many involved in cardiomyocyte proliferation and differentiation, including: dystroglycan 1 (*DAG1*, hyper- and hypomethylated; Supplementary Figure S2C), paired like homeodomain 2 (*PITX2*, hypomethylated; Supplementary Figure S2D), and Erb-B2 receptor tyrosine kinase 2 (*ERBB2*, hypomethylated) and 4 (*ERBB4*, hyper- and hypomethylated). Associations between intragenic methylation and gene expression are context-specific (Varley et al., 2013). Nevertheless, temperature-associated differential methylation within genes involved in heart development demonstrates the potential for corresponding differences in gene expression.

### EST Differential Methylation Impacts Binding Sites for TFs Involved in Cell Proliferation

As methylation is known to affect gene regulation through the alteration of TF binding sites, we sought to identify enriched motifs in the vicinity of DM CpGs between EST treatments. We submitted 100-bp sequences centered on the 2,175 DM CpGs for motif enrichment analysis, and were specifically interested in identifying motifs that were uniquely enriched among hyper- or hypomethylated sequences. We identified 71 motifs for 49 TFs uniquely enriched among hypermethylated regions, and 120 motifs for 81 TFs enriched among hypomethylated regions (Supplementary Table S4). Among the most uniquely enriched binding motifs for hypermethylated regions were numerous members of the zinc finger and BTB domain containing (ZBTB) family (ZBTB7C, ZBTB7A, ZBTB7B, and ZBTB49), many of which have been shown to negatively regulate cell proliferation (Jeon et al., 2014; Liu et al., 2014). Among hypermethylated regions, the most uniquely enriched binding motif was for musculin (MSC), which functions as a repressor of the myogenic factor *MYOD* (Zhao and Hoffman, 2006).

We submitted the gene IDs for TFs of hyper- and hypomethylated motifs to the PANTHER database for GO enrichment analysis. Uniquely enriched terms among TFs of hypermethylated motifs were not directly related to heart development or function; however, TFs of hypomethylated motifs were uniquely enriched for the GO terms “cardiovascular system development” and “positive regulation of cell population proliferation” (Table 3). Genes associated with these terms included several with known roles in positively regulating cardiomyocyte proliferation, including retinoic acid receptor alpha (*RARA*) (Xavier-Neto et al., 2015), T-box transcription factor 20 (*TBX20*) (Lu et al., 2017), lymphoid enhancer binding factor 1 (*LEF1*) (Ye et al., 2019) and *PITX2*, which was also found to be hypomethylated at 38.9°C. Overall, these results reveal the potential for decreased binding ability (via hypermethylation) of TFs involved in negatively regulating cell proliferation, and increased binding ability (via hypomethylation) of TFs involved in positively regulating cardiomyocyte proliferation at 38.9 vs. 37.8°C.

**TABLE 3:** Uniquely enriched GO terms among TFs of hypomethylated motifs.

**GO term**	**No. Genes**	**Enrichment**	***p*-value**
embryo development ending in birth or egg hatching	13	5.42	8.08E-07
vasculature development	9	4.77	1.20E-04
cardiovascular system development	9	4.68	1.38E-04
circulatory system development	12	3.79	7.37E-05
positive regulation of cell population proliferation	12	3.58	1.25E-04
cellular response to stress	17	2.67	1.59E-04

### CO_2_ Differential Methylation Impacts Genes Involved in Heart Development and Response to Hypoxia

We identified a total of 9,823 DM CpG between at least one pair of CO_2_ treatments. Among these, 3,652 were DM between 0.4 and 0.1% CO_2_ chicks, 4,695 between 0.8 and 0.4% CO_2_, and 4,482 between 0.8 and 0.1% CO_2_ (Table 4 and Supplementary Table S5). In each contrast, the higher CO_2_ treatment was associated with a higher proportion of hypermethylated CpGs, especially when comparing 0.8% CO_2_ to the other treatments.

**TABLE 4:** Summary of CO_2_ differential methylation analyses.

**Contrast**	**DM CpGs**	**Hypermethylated**	**Hypomethylated**	**Promoter hyper/hypo DMGs**	**Intragenic hyper/hypo DMGs**
0.4 vs. 0.1%	3652	1951	1701	124/106	524/517
0.8 vs. 0.4%	4695	2760	1935	165/156	744/514
0.8 vs. 0.1%	4482	2782	1670	176/119	671/457

DM genes were enriched for more heart- and muscle-specific GO terms when contrasting 0.8% CO_2_ to either of the other two CO_2_ treatments (Table 5 and Supplementary Table S6) Among hypermethylated intragenic-DMGs for each of the three contrasts, 18 such GO terms were enriched among the 0.8 vs. 0.1% contrast, while only five and six terms were enriched at 0.4 vs. 0.1% and 0.8 vs. 0.4%, respectively. Among hypomethylated intragenic-DMGs, the 0.8 vs. 0.4% CO_2_ contrast yielded the greatest number of enriched heart/muscle-related terms (*n* = 23), while the 0.4 vs. 0.1% and 0.8 vs. 0.1% yielded only three and seven, respectively. Intragenic-DMGs at 0.8% CO_2_ vs. 0.4% and 0.1% included: *PDLIM7*, whose resulting protein regulates valve annulus size and hemostasis (Krcmery et al., 2013); *TGFB1*, which regulates postnatal cardiomyocyte differentiation (Engelmann et al., 1992); and *ILK*, which has been shown to exhibit protective effects against cardiomyopathy (Hannigan et al., 2007). Additionally, we also identified an enrichment of DMGs associated with response to low oxygen only when comparing 0.8 to 0.1% CO_2_ (Table 5). Hypomethylated intragenic-DMGs were enriched for the GO terms “response to decreased oxygen levels” and “response to hypoxia,” and these were not enriched among the DMGs in the other two contrasts.

**TABLE 5:** Enriched GO terms among CO_2_ intragenic-DMGs related to heart/muscle development and hypoxic response.

**GO term**	**0.4 vs. 0.1 CO_2_**		**0.8 vs. 0.4 CO_2_**		**0.8 vs. 0.1 CO_2_**	
	**Enr.**	***P*-value**	**Enr.**	***P*-value**	**Enr.**	***P*-value**
**Intragenic-hypermethylated genes**						
positive regulation of striated muscle cell differentiation	5.3	2.3E-04			4.7	2.5E-04
positive regulation of striated muscle tissue development	4.5	6.5E-04			4.0	7.6E-04
positive regulation of muscle organ development	4.5	6.5E-04			4.0	7.6E-04
cardiac ventricle development	3.5	5.1E-04			3.2	3.5E-04
cardiac chamber development	3.1	4.7E-04	2.7	5.9E-04	2.8	5.2E-04
muscle organ development			2.5	3.4E-05	2.6	2.9E-05
muscle structure development			2.2	1.6E-05	2.4	5.2E-06
muscle tissue development			2.1	9.7E-04		
heart development			1.9	1.8E-04	2.0	1.3E-04
cardiovascular system development			2.6	5.3E-09	2.5	1.7E-07
positive regulation of myotube differentiation					5.6	1.2E-03
positive regulation of striated muscle cell differentiation					4.7	2.5E-04
positive regulation of muscle cell differentiation					4.3	5.7E-05
muscle tissue morphogenesis					3.9	4.4E-04
positive regulation of muscle tissue development					3.9	8.3E-04
muscle organ morphogenesis					3.6	8.0E-04
cardiac chamber morphogenesis					2.9	1.3E-03
cardiac muscle tissue development					2.7	1.2E-03
**Intragenic-hypomethylated genes**						
positive regulation of striated muscle tissue development	4.7	5.1E-04				
positive regulation of muscle organ development	4.7	5.1E-04				
mitral valve development			18.4	2.8E-05		
heart valve formation			13.5	4.8E-04		
pulmonary valve morphogenesis			10.1	1.2E-03		
atrioventricular valve development			8.4	5.8E-04		
aortic valve morphogenesis			8.1	6.9E-04		
heart valve morphogenesis			7.3	1.1E-05	6.5	2.0E-04
aortic valve development			7.2	1.1E-03		
cardiac atrium development			7.2	3.6E-04		
semi-lunar valve development			6.8	4.8E-04		
heart valve development			6.5	2.5E-05	5.8	3.7E-04
ventricular septum development			5.3	1.1E-04		
cardiac septum development			4.2	1.2E-04		
cardiac ventricle development			3.6	4.3E-04		
cardiac muscle tissue development			3.3	3.2E-04		
regulation of muscle tissue development			3.3	9.4E-04		
cardiac chamber development			3.2	3.9E-04		
heart morphogenesis			2.7	6.0E-04	3.0	1.5E-04
muscle tissue development			2.4	1.2E-03		
muscle structure development			2.3	2.5E-04		
heart development			2.2	1.5E-04	2.2	6.1E-04
cardiac chamber morphogenesis					3.7	6.6E-04
cardiovascular system development					2.7	2.5E-06
cellular response to oxidative stress					2.7	1.0E-03
response to hypoxia					2.7	1.1E-04
response to decreased oxygen levels					2.6	1.7E-04
response to oxygen levels					2.4	5.5E-04

### EWAS Identifies Methylation Signatures Significantly Associated With HW

**FIGURE 3:**
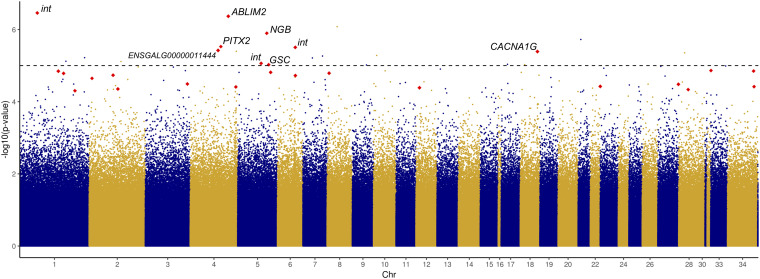
Manhattan plot displaying –log10 *p*-values of association between CpG methylation rate and log2-transformed relative heart weight, ordered by chromosome. Red points indicate DM CpGs; significantly associated and DM CpGs are labeled with their corresponding gene ID (int, intergenic).

Given the significant difference in HW between chicks of different incubation ESTs, we sought to identify CpG sites at which methylation rate was significantly associated with HW. We tested 1,521,346 CpG sites and identified 23 significant associations (Figure 3 and Supplementary Table S7), which represented a modest enrichment in significant *p*-values compared to a random distribution (Supplementary Figure S3). Three of these CpGs were within gene promoter regions (in *GFI1*, *SP9*, and *ST6GALNAC4*), 13 were within gene bodies (exons or introns), and seven were in intergenic regions.

Among the 23 significantly associated CpG sites, nine were also significantly DM between EST treatments, and only one site – the hypomethylated CpG in *PITX2* – had a methylation difference greater than 10%. This site was found to be significantly positively correlated with HW (*r* = 0.60, *p* = 2.97E-06), along with four other sites: two exonic CpGs in neuroglobin (*NGB*) and goosecoid homeobox (*GSC*), an intronic CpG in calcium voltage-gated channel subunit alpha1 G (*CACNA1G*), and an intergenic CpG. Four hypermethylated CpGs were negatively associated with HW: an exonic CpG in *ENSGALG00000011444*, an intronic CpG in actin binding LIM protein family member 2 (*ABLIM2*), and two intergenic CpGs.

### RT-qPCR Identifies Genes With Coordinated Differences in Gene Methylation and Expression Between EST Treatments

**FIGURE 4:**
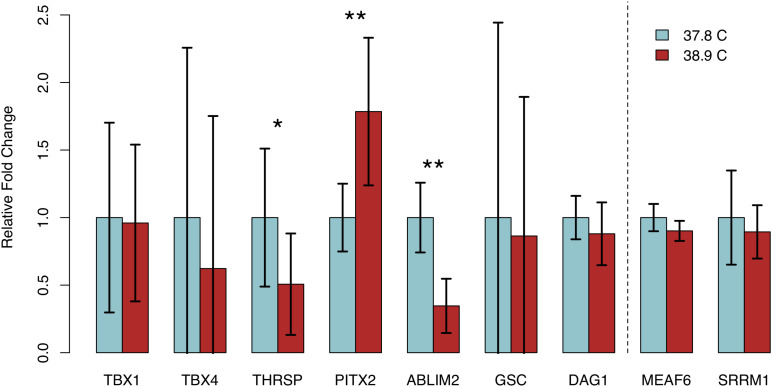
Relative fold changes in transcript abundance (38.9°C relative to 37.8°C) for seven expressed genes exhibiting differential methylation and two stably methylated genes between temperature treatments. Error bars indicate standard deviation. *p < 0.05, **p < 0.01.

We assessed transcript abundance of eight genes exhibiting differential methylation and/or significant associations with HW via RT-qPCR, to determine if differential methylation was associated with differences in gene expression. We detected quantifiable transcript abundance for all genes except *NGB*, which was undetectable in multiple samples in both EST treatments. Three genes – *ABLIM2*, *PITX2*, and *THRSP* – exhibited significant differences in transcript abundance between EST treatments (Figure 4). In all three cases, abundance was higher in the treatment with lower methylation. We observed significantly lower *THRSP* abundance in 38.9°C chicks (*p* = 0.046), which is consistent with the promoter hypermethylation observed. Abundance of *PITX2*, which contained a hypomethylated CpG in its last exon, was significantly higher in 38.9°C chicks (*p* = 7.59E-03). *ABLIM2*, containing a hypermethylated intronic CpG, exhibited lower abundance at 38.9°C (*p* = 1.37E-03). Among the remaining five DMGs, the differences in expression were negatively associated with the differences in methylation for *DAG1*, while the differences were positively associated for *TBX1*, *TBX4*, and *GSC*. We also assessed transcript abundance in two genes that did not exhibit differential methylation (*MEAF6* and *SRRM1*), and did not detect differences in abundance between ESTs. These results have identified candidate genes exhibiting coordinated differences in methylation and expression in the developing chick heart that are associated with incubation EST.

## Discussion

To the best of our knowledge, this study represents the largest epigenome-wide analysis in livestock species, the first EWAS in broiler chickens, and the first study assessing genome-wide DNA methylation between chickens incubated in different environments. RRBS was performed in cardiac tissue collected from broiler chicks in a study that recapitulated the established negative correlation between incubation EST and HW. We observed significantly lower HW in chicks incubated at 38.9 vs. 37.8°C, which was independent of differences in incubation length. The heart appears to be uniquely impacted by incubation EST, a finding that has been observed in numerous studies showing up to 30% lower HW in 38.9°C chicks with no significant differences in organs such as the liver, intestines or stomach (Molenaar et al., 2011; Maatjens et al., 2014, 2016; Nangsuay et al., 2016).

Using RRBS, we assessed methylation at over 3 million CpGs across 84 heart samples. Global methylation rates in these samples were low (13.8–20.4%), due to the fact that a majority of sites exhibited methylation rates less than 10%. RRBS reads are disproportionally derived from CpG dense regions of the genome, which are known to be less methylated than the genome at large (Meissner et al., 2008). However, RRBS data from eight pig tissues revealed an average methylation rate of 41% (Schachtschneider et al., 2011). Thus the low methylation observed in our data may be specific to the species, tissue, or stage of development. To date, whole-genome bisulfite sequencing studies in *G. gallus* and other avian species have reported global CpG methylation rates between 50 and 65% (Li et al., 2015; Derks et al., 2016; Lee et al., 2017; Zhang et al., 2017, 2018), which is lower than the 60–80% that has been reported in mammalian studies. It is possible that the low levels of CpG methylation detected in our data can be attributed to lower CpG methylation rates in avian genomes.

We identified thousands of DM CpGs between ESTs, and annotation of DMGs revealed an enrichment for general and heart-specific developmental processes. While promoter-DMGs were only significantly enriched for processes related to general development, many of these genes are known to be involved in heart development. Among these were two T-box transcription factors, *TBX1* and *TBX4*, that were promoter-hypomethylated at 38.9°C. *TBX1* has been shown to promote cell proliferation and inhibit differentiation of heart cells (Xu et al., 2004; Chen et al., 2009). *TBX4* has been shown to be expressed in the developing heart, and has been hypothesized to play a role in regulating tissue-specific gene expression (Krause et al., 2004). Also among promoter-DMGs were two genes involved in response to thyroid hormone (*THRA*, hypomethylated and *THRSP*, hypermethylated at 38.9°C), which has previously been hypothesized to mediate temperature-driven differences in heart growth. *THRA* is the major thyroid hormone receptor expressed in the heart (Gloss et al., 2001), and *THRA* mutations in zebrafish have been linked to defects in heart development (Marelli et al., 2017). *THRSP* is expressed in chicken cardiac tissue (Ren et al., 2017), and research has shown that overexpression of *THRSP* results in decreased levels of genes involved in heart development (Wellberg et al., 2014). Intragenic-DMGs were enriched for GO terms related to heart development. Among these, *DAG1* possessed hypo- and hypermethylated CpGs, and encodes a glycoprotein that has been shown to play an important role in the inhibition of cardiomyocyte proliferation in mice (Morikawa et al., 2017). *PITX2* contained an exonic CpG that was significantly hypomethylated at 38.9°C. This gene encodes a homeobox TF with roles in heart development that have been reviewed extensively (Franco et al., 2017); of note, it has been shown to promote cardiomyocyte proliferation in mice (Tao et al., 2016). *ERBB2* and *ERBB4* encode receptor kinases that have been shown to promote cardiomyocyte proliferation and survival (Zhao et al., 1998). The observed differential epigenetic regulation of genes involved in cardiomyocyte proliferation and differentiation may be contributing to observed differences in HW between chicks of different incubation ESTs.

Furthermore, assessment of enriched binding motifs among DM loci provides evidence that temperature-differential methylation is impacting the developmental trajectories of the chick heart. Methylation is known to reduce the binding potential of TFs by altering binding motif sequences (Yin et al., 2017) At 38.9°C, hypomethylated binding motifs – which would be predicted to be more accessible due to lower methylation levels – were enriched for TFs involved in positive regulation of cardiomyocyte proliferation. Conversely, the binding sites for TFs involved in negative regulation of cell proliferation – namely TFs in the ZBTB family – were the most enriched motifs among hypermethylated regions, which would be expected to be less accessible. The more favorable binding of pro-proliferation TFs in the cardiac methylome of chicks at higher temperatures suggests that observed differences in HW may be associated with differences in rates of differentiation and maturation.

We also identified thousands of DM CpGs between CO_2_ treatments. Increasing CO_2_ concentrations above 0.8% during late stages of incubation have benefits in commercial settings such as reduced hatching time variability (van Roovert-Reijrink, 2016). However, continuous incubation in a hypercapnic environment has been shown to increase ascites incidence later in life (Hassanzadeh et al., 2002). Not only did differential methylation at 0.8% CO_2_ relative to the lower treatments disproportionately affect genes involved in heart development, but contrasting 0.8% with 0.1% CO_2_ revealed differential methylation of genes that were enriched for hypoxia response GO terms. Such DMGs associated with hypoxia included: *ETS1*, a TF that has been shown to activate hypoxia-inducible genes (Salnikow et al., 2008); *SRC*, encoding a tyrosine kinase that is upregulated in rat cardiomyocytes by hypoxia to activate MAPK signaling pathways (Seko et al., 1996); and *DNM2*, encoding a GTPase and actin-binding protein that has been shown to be downregulated in cardiomyocytes subject to hypoxic conditions (Shi et al., 2014). Hypoxia-inducible genes being specifically overrepresented when comparing extremes for CO_2_ indicate a cardiac response to differences in gas exchange via differences in gene regulation. Incubation CO_2_ level was not associated with differences in HW in this study, which has been shown previously (Maatjens et al., 2014). However, CO_2_ concentration may have observable effects on physiological processes that were not assessed here – including angiogenesis – that may contribute to adverse conditions later in life including increased ascites risk.

Our EWAS revealed a small but significant enrichment of association *p*-values between CpG methylation and HW, suggesting a relationship between gene regulation in the heart and its size at hatch. At the 23 significantly associated CpGs, epigenetic regulation of gene expression by DNA methylation may directly or indirectly affect heart growth. A DM CpG in an exon of *PITX2* was also the most significantly positively correlated with HW. We identified additional significant CpGs in our EWAS that were also DM, albeit at a difference less than 10%. An intragenic CpG in *ABLIM2* was significantly hypermethylated at 38.9°C and negatively correlated with HW; this gene encodes a actin binding protein that has been shown to be highly expressed in striated muscle and is thought to regulate cytoskeletal signaling pathways (Barrientos et al., 2007). Additionally, an intergenic CpG in *NGB* was significantly hypomethylated and positively correlated with HW. The gene, encoding neuroglobin, was found to be differentially expressed in response to water temperature in Whiteleg shrimp (Wu et al., 2017). However, we did not detect NGB transcript abundance at appreciable levels in our samples, suggesting a limited role at this stage in chick heart development and temperature response. CpGs in these genes, along with those identified in other genes and in intergenic regions, are strong candidates for identifying epigenetic-mediated associations between temperature and heart development.

We identified coordinated changes in methylation and expression for three of the eight tested genes: *ABLIM2*, *PITX2*, and *THRSP*. In all three cases, direction of the difference in gene expression was opposite that of the difference in methylation between ESTs. This inverse relationship was expected at *THRSP*, since the DM CpG was immediately upstream of the TSS in the likely promoter region. Intragenic methylation in *ABLIM2* and *PITX2* were also negatively associated with expression, and this is in contrast to findings in other animal studies that the majority of CpGs in such regions are positively correlated with gene expression. Cases of intragenic methylation being negatively associated with gene expression have been reported by ENCODE (Varley et al., 2013), and they found these CpGs to be enriched in intragenic enhancers. The presence of CpGs in *ABLIM2* and *PITX2* at which methylation rate is inversely correlated with gene expression suggests that these CpGs may be located within gene regulatory elements. To explore this idea, we searched for binding motifs in the genomic regions flanking in DM CpGs in *ABLIM2* and *PITX2*. The *PITX2* DM regions had strong sequence similarity to the Kruppel Like Factor 5 (*KLF5*) binding motif, and this TF has been shown to be upregulated during heat stress in chickens previously (Sun et al., 2015). Additionally, the *ABLIM2* DM regions showed strong sequence similarity to the binding motif of Achaete-Scute Family BHLH Transcription Factor 2 (*ASCL2*), which is a known inhibitor of myogenic differentiation (Wang et al., 2017). While differential expression was not observed for five of the eight tested DMGs, this could be due in part to the limited number of samples that were assayed via qPCR (*n* = 6/trt) relative to the number assayed via RRBS (*n* = 42/trt).

Among these three DM and differentially expressed genes, *PITX2* has the most well-established role in heart development, specifically in promoting cardiomyocyte proliferation. In this study, increased EST was associated with both decreased methylation at an exonic CpG in *PITX2* as well as increased *PITX2* transcript abundance. As *PITX2* is known to promote cardiomyocyte proliferation, this suggests an increased proliferative capacity in the hearts of 38.9°C chicks. This conflicts with results reported in Romanoff, 1960, in which increased incubation temperature was associated with a reduced mitotic index from E8 to hatch. However, our results indicate that proliferation in high EST chicks may be increased relative to lower EST chicks as a partial compensatory mechanism immediately following hatch, and that developmental trajectories of hearts at different ESTs continue to be affected even after incubation.

In conclusion, this study has identified differential methylation patterns in the post-hatch chick heart associated with differences in incubation EST and CO_2_ level, and such differences may be impacting heart growth and development through associated changes in TF binding and gene expression. Future studies should seek to further assess differences in methylation between incubation treatments at later stages of post-hatch life. Additionally, characterizing epigenetic patterns in additional organs responsible for regulating body temperature, including the hypothalamus and thyroid gland, may help in characterizing the mechanisms regulating temperature-driven differences in heart development. Knowledge of such epigenetic signatures influenced by early life environment may benefit animal breeding by serving as predictors of future animal performance.

## Data Availability Statement

The RRBS data is available by the following SRA/ENA accession number PRJEB39999.

## Ethics Statement

The animal study was reviewed and approved by the Central Animal Experiments Committee (The Hague, Netherlands; approval no. 2016.W-0087).

## Author Contributions

OM, MG, and RPC conceived of the study design. HB conducted the incubation study from which heart samples were collected. RJC performed all of the bioinformatics, differential methylation, GO enrichment analyses, as well as the EWAS, and wrote the manuscript. MP and CE contributed intellectually to the interpretation of results. All authors read and approved of the manuscript.

## Conflict of Interest

The authors declare that the research was conducted in the absence of any commercial or financial relationships that could be construed as a potential conflict of interest.

## Footnotes

1 https://github.com/nugentechnologies/NuMetRRBS
