# Supplementary figures and images for: Genome-Wide Assessment of DNA Methylation in Chicken Cardiac Tissue Exposed to Different Incubation Temperatures and CO2 Levels
Source: Front Genet. 2020 Oct 28;11:558189. doi: 10.3389/fgene.2020.558189 (PMC7655987; doi:10.3389/fgene.2020.558189)

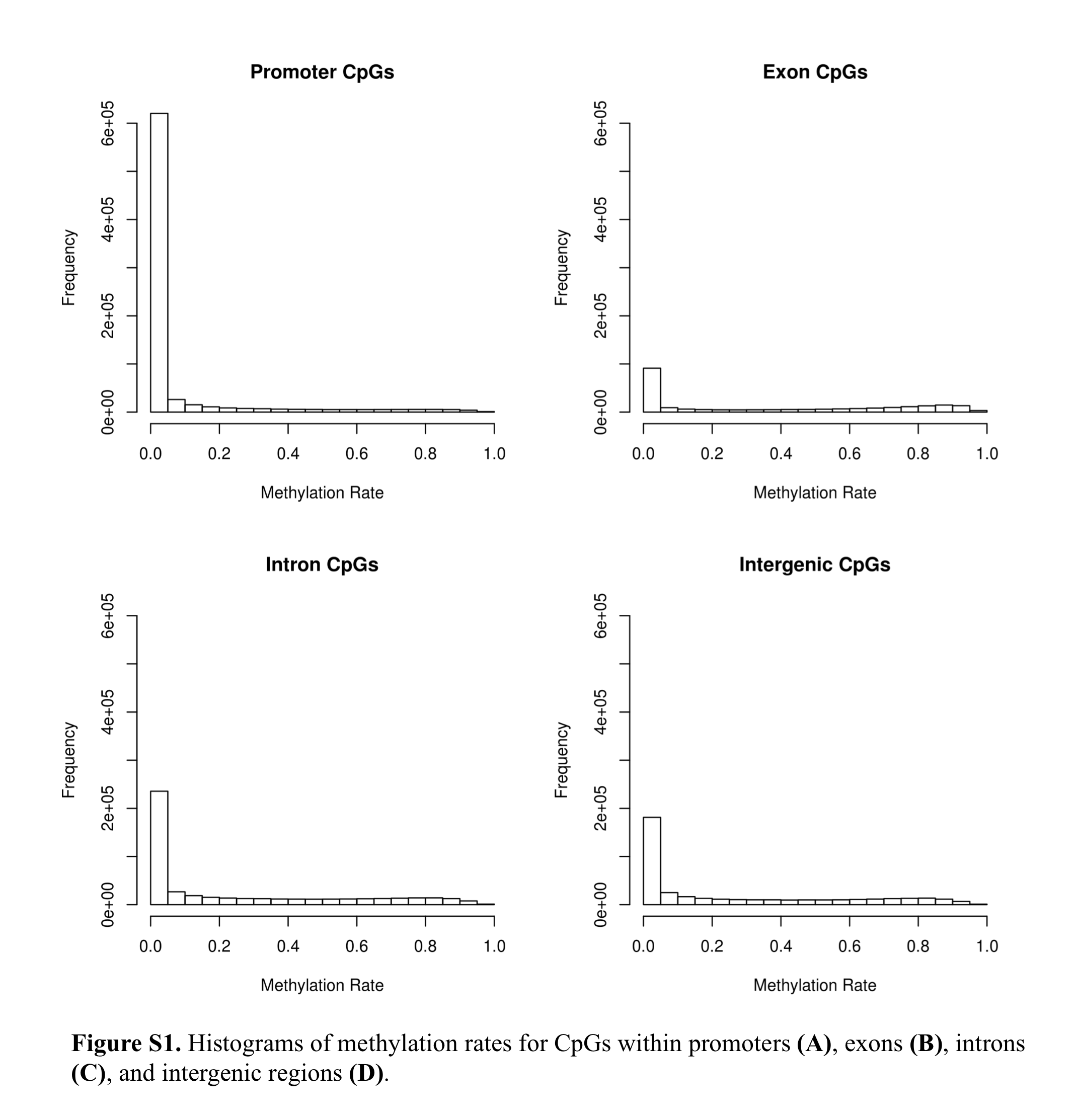

Supplement: Supplementary file 1 [file Image_1.TIFF]

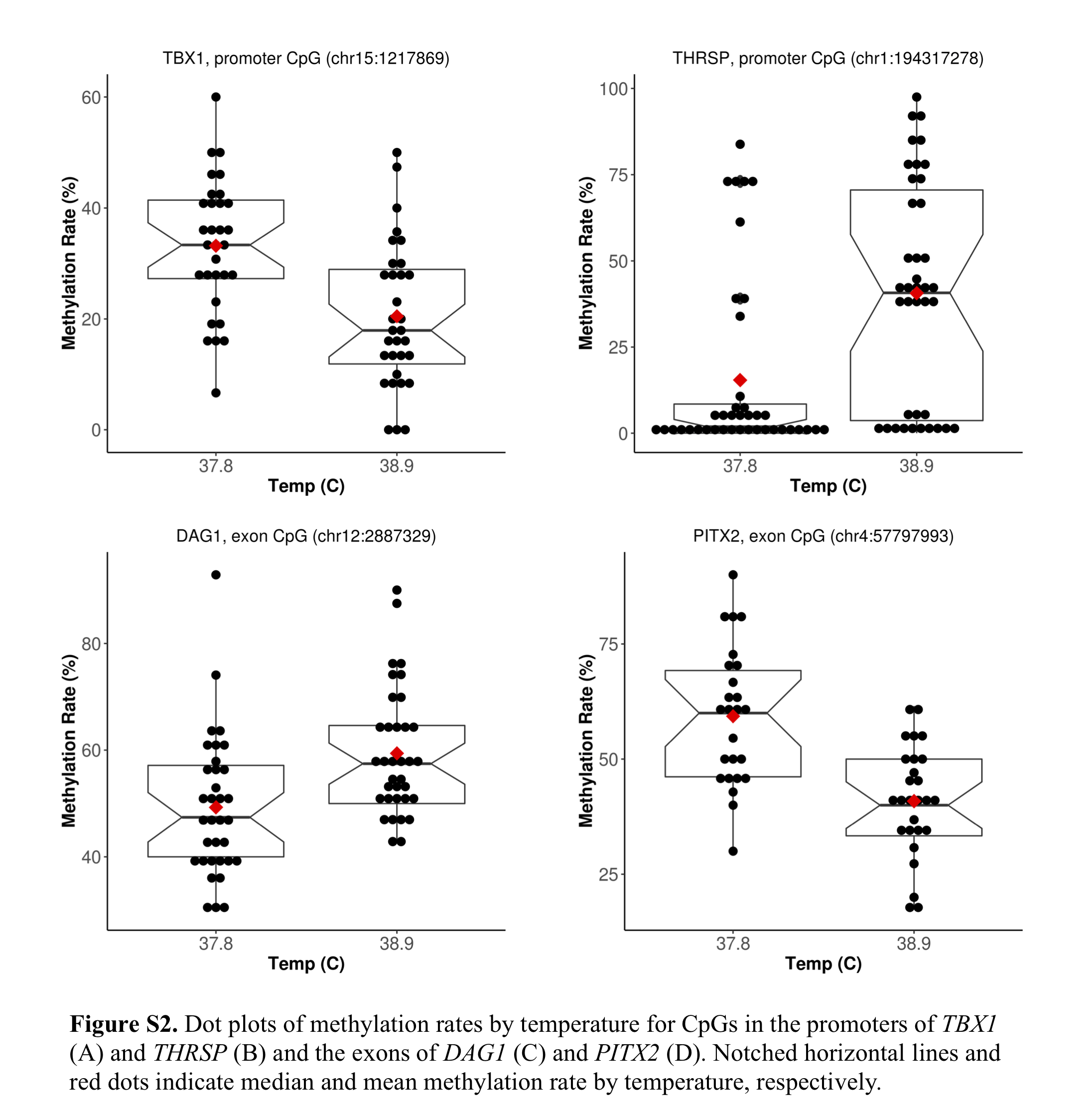

Supplement: Supplementary file 2 [file Image_2.TIFF]

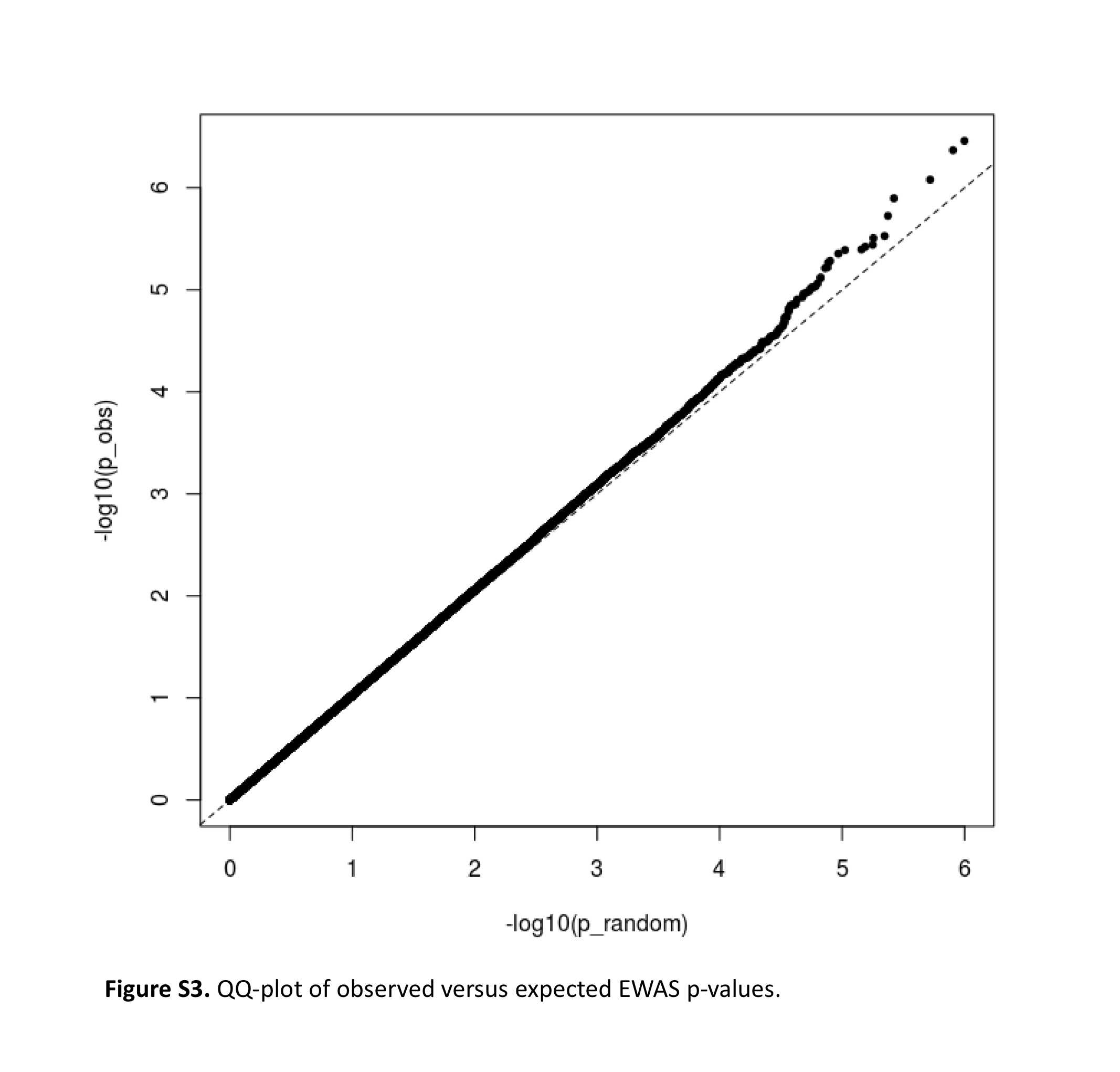

Supplement: Supplementary file 3 [file Image_3.TIFF]
